# Prediction of Clinical Outcome at Discharge After Rupture of Anterior Communicating Artery Aneurysm Using the Random Forest Technique

**DOI:** 10.3389/fneur.2020.538052

**Published:** 2020-10-29

**Authors:** Nengzhi Xia, Jie Chen, Chenyi Zhan, Xiufen Jia, Yilan Xiang, Yongchun Chen, Yuxia Duan, Li Lan, Boli Lin, Chao Chen, Bing Zhao, Xiaoyu Chen, Yunjun Yang, Jinjin Liu

**Affiliations:** ^1^Department of Radiology, The First Affiliated Hospital of Wenzhou Medical University, Wenzhou, China; ^2^Department of Ultrasonography, The First Affiliated Hospital of Wenzhou Medical University, Wenzhou, China; ^3^Department of Neurosurgery, Renji Hospital, Shanghai Jiaotong University School of Medicine, Shanghai, China

**Keywords:** aneurysms, subarachnoid hemorrhage, random forest, prediction, outcome

## Abstract

**Background:** Aneurysmal subarachnoid hemorrhage (SAH) is a devastating disease. Anterior communicating artery (ACoA) aneurysm is the most frequent location of intracranial aneurysms. The purpose of this study is to predict the clinical outcome at discharge after rupture of ACoA aneurysms using the random forest machine learning technique.

**Methods:** A total of 607 patients with ruptured ACoA aneurysms were included in this study between December 2007 and January 2016. In addition to basic clinical variables, 12 aneurysm morphologic parameters were evaluated. A multivariate logistic regression analysis was performed to determine the independent predictors of poor outcome. Of the 607 patients, 485 patients were randomly selected for training and the remaining for internal testing. The random forest model was developed using the training data set. An additional 202 patients from February 2016 to December 2017 were collected for externally validating the model. The prediction performance of the random forest model was compared with two radiologists.

**Results:** Patients' age (odds ratio [OR] = 1.04), ventilated breathing status (OR = 4.23), World Federation of Neurosurgical Societies (WFNS) grade (OR = 2.13), and Fisher grade (OR = 1.50) are significantly associated with poor outcome. None of the investigated morphological parameters of ACoA aneurysm is an independent predictor of poor outcome. The developed random forest model achieves sensitivities of 78.3% for internal test and 73.8% for external test. The areas under receiver operating characteristic (ROC) curve of the random forest model were 0.90 for the internal test and 0.84 for the external test. Both sensitivities and areas under ROC curves of our model are superior to those of two raters in both internal and external tests.

**Conclusions:** The random forest model presents good performance in predicting the outcome after rupture of ACoA aneurysms, which may aid in clinical decision making.

## Introduction

Subarachnoid hemorrhage (SAH) is found in ~5% of all strokes (1). The primary cause of SAH is the rupture of an intracranial aneurysm, which accounts for ~85% of all cases (1). Anterior communicating artery (ACoA) aneurysm is the most frequent location of intracranial aneurysms (2); ~40% of aneurysmal SAH are attributed to ACoA aneurysms (3). Aneurysmal SAH is a devastating disease and has high mortality and morbidity. The study of the International Subarachnoid Aneurysm Trial (4) shows that 25.4% patients allocated to endovascular treatment and 36.4% patients allocated to neurosurgery were dependent or dead at 2 months. The diagnosis and acute management of aneurysmal SAH represents a challenge to clinicians (1, 5). Accurately determining the prognosis after aneurysmal SAH is crucial for providing adequate information to patients' family, guiding treatment options, and detecting subgroups of patients to be beneficial from certain treatments (6, 7).

Outcome after aneurysmal SAH is associated with many factors (7, 8). Different clinical scales have been applied to classify aneurysmal SAH, such as Federation of Neurological Surgeons (9) and Fisher grade (10). It remains challenging to accurately predict the outcome after aneurysmal SAH because decision making is driven largely by the clinician's experience and instinct, which may result in significant variability among different clinicians due to the complexity of aneurysmal SAH patients. Machine learning can reduce this variability among clinicians and is capable of finding complex relationships in big data and rapidly analyzing many variables to predict patients' outcomes of interest. It has been successfully applied in clinical prognosis analysis (11, 12).

In this exploratory study, we aim to predict the outcome at discharge after rupture of ACoA aneurysm using a random forest machine learning technique.

## Methods

### Study Design and Patients

This study was approved by the institutional ethics committee. A total of 773 consecutive patients with ACoA aneurysms were admitted to our hospital between December 2007 and January 2016. These patients were screened from the electronic medical record system by searching the keywords “aneurysm” and “anterior communicating artery.” Patients with unruptured aneurysms or with fusiform aneurysms were excluded from this study. Those without outcome information were also excluded from this study.

### Image Acquisition and Aneurysm Morphologic Measurement

All patients underwent computed tomography angiography (CTA) examinations. Matrix size of DICOM images was 512X512. Morphological parameters were measured from CTA images after volume rendering reconstruction. Scanning was performed on a 320-detector row CT scanner (Aquilion ONE, Toshiba Medical Systems, Tochigi, Japan), a 64-channel multidetector CT scanner (LightSpeed VCT 64, GE Medical Systems, Milwaukee, WI, USA), or a 16-channel multidetector CT scanner (LightSpeed pro 16, GE Medical Systems). Non-ionic contrast agent, Iopromid with 300 mg/ml iodine, was injected via an antecubital vein with a high pressure injector at 3.0 ml/s (1.0–2.0 ml/kg) for the 16-channel multidetector CT; non-ionic contrast agent, Iodixanol 320 or Iohexol 350, was injected via an antecubial vein with a high pressure injector at 4.0 ml/s (0.8–1.0 ml/kg) for the 64-channel multidetector CT or 320-detector rows CT.

Twelve aneurysm morphologic parameters were evaluated, including aneurysm projection, A1 segment configuration, aneurysm size, vessel size, aneurysm height, perpendicular height, neck size, aspect ratio, size ratio, aneurysm angles, vessel angle, and flow angle. These parameters have been defined elsewhere (13–15). For completeness and the reader's convenience, we provide detailed aneurysm sizes and angle measurements in Supplementary Figure 1.

### Data Collection

In addition to aneurysm morphologic parameters, the following patients' data were collected: patients' sex and age, medical history (including hypertension, current smoking, coronary artery disease, and previous stroke), Glasgow coma score (GCS) and World Federation of Neurosurgical Societies (WFNS) grade before treatment, Fisher grade, treatment methods, and outcome at discharge.

### Outcome Assessment

Clinical outcome at discharge was evaluated using Glasgow Outcome Scale (GOS) (16) by independent radiologists who did not participate in the treatment. A GOS of one indicates death, two represents persistent vegetative state, and three means severe disability (conscious but disabled). Patients with moderate disability (disabled but independent) and recovery were defined as a GOS of 4 and 5, respectively. Poor outcome was defined as a GOS of 1 or 2 or 3.

### Random Forest

Random forest is an ensemble and supervised learning algorithm (17, 18), which builds multiple decision trees and merges them together to obtain a more stable and accurate prediction. It can be used for solving both classification and regression problems. Moreover, it is capable of measuring the relative importance of each feature for prediction. It is a popular machine learning technique because of its good accuracy, robustness, and ease of use, and it has been widely applied in biomedical science (19–23). We used a random forest machine learning technique to predict the clinical outcome after rupture of ACoA aneurysm in this study. Of the included 607 patients, 485 random patients (80%) were selected for training and the remaining 122 patients for testing. Correlation-based feature selection method was applied for feature selection using the training dataset. Patients' age, breathing status, pupillary reactivity, WFNS grade, and Fisher grade were selected. The grid search method was used for hyper-parameter optimization, and a 10-fold cross-validation strategy was adopted during training. In the random forest model, the number of trees in the forest was set to 10, the maximum depth of the tree was determined to be three, and the number of features to consider when looking for the best split was set to four. The performance of the random forest model was evaluated by the accuracy, sensitivity, specificity, and area under receiver operating characteristic (ROC) curve. Sensitivity is the percentage of patients with poor outcome who are correctly predicted as such. Specificity measures the proportion of patients with favorable outcomes who are correctly predicted as such. In this study, the open source data mining software Weka 3.8.1 was used for feature selection; Python 2.7 was the coding language; the free machine learning library scikit-learn 2.0 was applied for random forest model development (24, 25).

### Additional Independent Testing Data Set

To validate the developed random forest model, we further retrospectively collected patients with ruptured ACoA aneurysm in our hospital from February 2016 to December 2017. This data set was not used for random forest modeling and only used for additional independent model testing.

### Clinical Outcome Assessment by Radiologists

Clinical outcome assessments for both internal and external tests were independently performed by two radiologists who were blind to the clinical outcome at discharge. The assessments relied on the identical baseline data in the two testing data sets. The performance of the assessments from the two raters was evaluated by accuracy, sensitivity, specificity, and area under ROC curve.

### Statistical Analyses

Continuous variables were described as the mean±standard deviation and categorical variables as the frequency (percentage). Univariate analysis was performed to assess the relationship between these variables and patients' prognostic outcome at discharge. A *P* < 0.05 is considered statistically significant. We used patients' clinical variables and neurological findings at admission to predict clinical outcome at discharge. All statistically significant variables (*P* < 0.05) except treatment method in the univariate analysis were entered the multivariate regression analysis. A forward step-wise multivariate logistic regression analysis was conducted to determine the independent predictors of poor outcome. Assessment agreement between two raters was evaluated using Cohen's kappa statistic. All statistical analyses were performed by using IBM SPSS Version 22.0 (IBM SPSS, Armonk, New York, USA).

## Results

### Patient Characteristics

A total of 607 consecutive patients after rupture of ACoA aneurysm were enrolled in this study between December 2007 and January 2016 at our institution. Of the included patients, the mean age was 55.7 ± 12.0 years; 287 (47.3%) patients were men; 116 (19.1%) patients had a poor outcome at discharge, including 41 of 230 (17.8%) patients receiving surgical treatment, 30 of 291 (10.3%) patients receiving endovascular treatment, and 45 of 86 (52.3%) patients under conservative treatment (Table 1). Compared with patients with surgical treatment, those who underwent endovascular treatment were more likely to have a good outcome (*P* = .013).

**Table 1:** Baseline characteristics.

**Variables**	**Good outcome (*n* = 491)**	**Poor outcome (*n* = 116)**	**OR 95% CI**	***P*-value**
**Demographic**				
Men	237 (48.3%)	50 (43.1%)	0.81 (0.54–1.22)	0.317
Age (yr)	54.7 ± 11.6	59.8 ± 12.9	1.04 (1.02–1.06)	<0.001
**Medical history**				
Hypertension	239 (48.7%)	68 (58.6%)	1.49 (0.99–2.25)	0.055
Current smoking	152 (31.0%)	39 (33.6%)	1.13 (0.74–1.74)	0.579
Coronary artery disease	4 (0.8%)	2 (1.7%)	2.14 (0.39–11.81)	0.384
Previous stroke	12 (2.4%)	8 (6.9%)	2.98 (1.18–7.41)	0.021
**Clinical examination**				
Breathing status				<0.001
Spontaneous	485 (98.8%)	85 (73.3%)	1.0 (Referent)	
Ventilated	6 (1.2%)	31 (26.7%)	29.48 (11.94–72.80)	
Pupillary reactivity				<0.001
Reactive (at least unilaterally)	480 (97.8%)	77 (66.4%)	1.0 (Referent)	
Unreactive	11 (2.2)	39 (33.6%)	22.10 (10.86–45.00)	
**Neurological examination**				
GCS	14.4 ± 1.6	10.2 ± 4.4	0.66 (0.61–0.71)	<0.001
WFNS grade	1.4 ± 0.9	3.3 ± 1.7	2.46 (2.11–2.87)	<0.001
**Radiological findings**				
Fisher grade	3.0 ± 1.2	3.7 ± 0.8	2.30 (1.67–3.17)	<0.001
Multiple aneurysm	78 (15.9%)	18(15.5%)	1.18 (0.78–1.78)	0.436
Aneurysm size (mm)	5.3 ± 2.6	5.7 ± 2.5	1.07 (0.99–1.16)	0.111
Vessel size (mm)	1.9 ± 0.5	2.0 ± 0.5	1.33 (0.89–2.00)	0.166
Aneurysm height (mm)	4.2 ± 2.2	4.8 ± 2.3	1.12 (1.02–1.23)	0.013
Perpendicular height (mm)	3.4 ± 1.7	4.0 ± 2.0	1.20 (1.08–1.34)	0.001
Neck size (mm)	3.0 ± 1.2	3.2 ± 1.2	1.09 (0.92–1.30)	0.313
Aspect ratio	1.2 ± 0.6	1.4 ± 0.8	1.51 (1.13–2.02)	0.006
Size ratio	2.4 ± 2.1	2.7 ± 1.9	1.05 (0.96–1.15)	0.289
Aneurysm angle	70.0 ± 18.4	69.6 ± 18.6	1.00 (0.99–1.01)	0.834
Vessel angle	59.7 ± 27.2	60.4 ± 26.5	1.00 (0.99–1.01)	0.787
Flow angle	134.5 ± 28.3	136.2 ± 25.8	1.00 (1.00–1.01)	0.568
Aneurysm projection^a^				0.150
Anterior	345 (70.3%)	74 (63.8%)	1.0 (Referent)	
Posterior	117 (23.8%)	35 (30.2%)	1.40 (0.89–2.20)	
A1 segment configuration^a^				0.077
Symmetric	155 (31.6%)	34 (29.3%)	1.0 (Referent)	
Dominant	198 (40.3%)	38 (32.8%)	0.88 (0.53–1.45)	
Complete	109 (22.2%)	37 (31.9%)	1.55 (0.91–2.62)	
**Treatment methods**				<0.001
Endovascular treatment	261 (53.2%)	30 (25.9%)	1.0 (Referent)	
Surgical treatment	189 (38.5%)	41 (35.3%)	1.89 (1.14–3.13)	
Conservative treatment	41 (8.4%)	45 (38.8%)	9.55 (5.42–16.84)	

*SAH, subarachnoid Hemorrhage; GCS, Glasgow coma score; WFNS, World Federation Neurosurgical Societies*.

a *36 missing values*.

In the additional independent testing data set, there were 104 male patients and 98 female patients; their mean age was 56.8 ± 12.0 years. Of these patients, 160 (79.2%) had a favorable outcome, and 42 (20.8%) had a poor outcome; 54 patients received surgical treatment, 109 patients underwent endovascular treatment, and 29 patients received conservative treatment. More detailed characteristics of these patients are illustrated in Supplementary Table 1.

### Predictors of Poor Outcome in Univariate and Multivariate Analyses

Table 1 shows the univariate analyses of the association between clinical variables and poor outcome. Table 2 demonstrates the independent predictors of poor outcome determined by using a multiple logistic regression analysis. In univariate analysis, older age (*P* < 0.001), ventilated breathing status (*P* < 0.001), unreactive pupillary response (*P* < 0.001), lower GCS, higher WFNS grade (*P* < 0.001), higher Fisher grade (*P* < 0.001), and treatment methods (*P* < 0.001) were associated poor outcome. Two aneurysm morphologic parameters, including aneurysm height and aspect ratio, were significant larger in the patients with poor outcome than those with favorable outcome in univariate analyses; however, none of them showed significant difference in multivariate analysis. The multivariate analysis revealed four independent predictors of poor outcome: age (odds ratio [OR], 1.04; *P* = 0.001), ventilated breathing status (OR, 4.23; *P* = 0.01), WFNS grade (OR, 2.13; *P* < 0.001), and Fisher grade (OR, 1.50; *P* = 0.001).

**Table 2:** Results of multivariate logistic regression analysis.

**Variable**	**β coefficient^†^**	**OR**	**95% CI**	***P*-value**
Age	0.04 ± 0.01	1.04	1.02–1.06	0.001
Breathing status				0.01
Spontaneous		1.0 (Referent)		
Ventilated	1.44 ± 0.56	4.23	1.41–12.65	
WFNS grade	0.76 ± 0.10	2.13	1.76–2.58	<0.001
Fisher grade	0.41 ± 0.16	1.50	1.10–2.05	0.001

*OR, odds ratio; CI, confidence interval; WFNS, World Federation of Neurosurgical Societies*.

† *Values are means ± standard errors*.

### Comparison of Performance Between Random Forest Model and Two Raters

**Figure 1:**
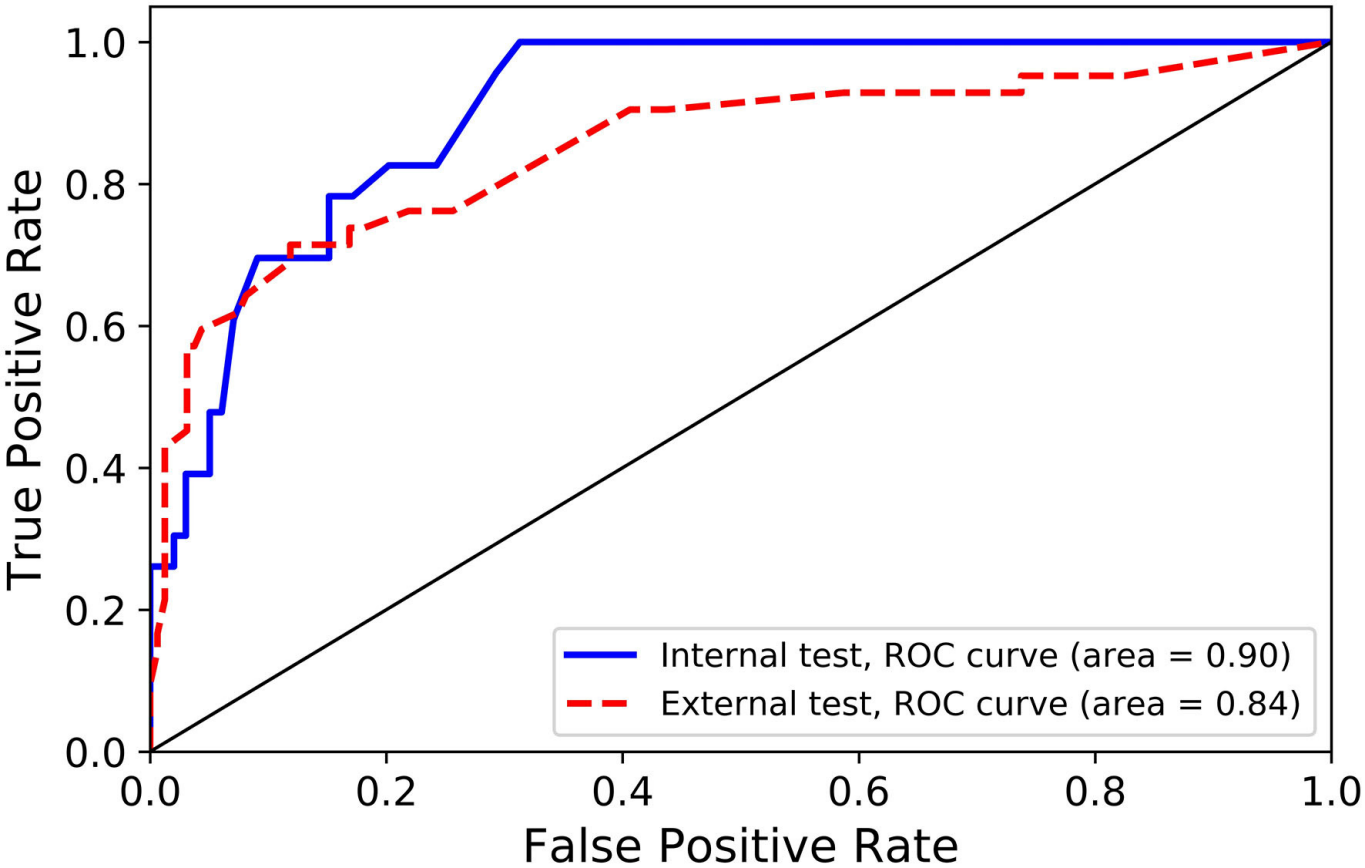
Receiver operating characteristic (ROC) curves for internal and external independent tests.

The performance of the random forest model and two raters in predicting clinical outcome are listed on Table 3. For the random forest model, 18 of the 23 poor cases were correctly predicted as poor, and 82 of 99 favorable cases were predicted as favorable in the internal test, and corresponding sensitivity and specificity were 78.3% (18/23) and 82.8% (82/99), respectively; in the external test, the prediction sensitivity was 73.8% and specificity was 83.1%. Figure 1 represents the ROC curves for the random forest model. The areas under the ROC curves were 0.90 for the internal test and 0.84 for the external test, which indicate that the prediction performance of the developed random forest model is good. The Cohen's kappa coefficient is 0.94, which indicates that the amount of agreement between the two radiologists is high. The specificities of the random forest model in the internal and external tests are inferior to those of the two raters; however, the sensitivities of the random forest model are higher than those of two raters in both internal and external tests, especially in the internal test (78.3% vs. 52.2 and 78.3% vs. 66.7% in the internal test; 73.8% vs. 66.7 and 73.8 vs. 66.7% in the external test), and the areas under the ROC curves of the random forest model are also superior to those of two raters in both internal and external tests (0.90 vs. 0.73 and 0.90 vs. 0.75 in the internal test; 0.84 vs. 0.78 and 0.84 vs. 0.78 in the external test).

**Table 3:** Comparison of performance between random forest model and two raters.

**Actual outcome**	**Predicted outcome**			Area under ROC^*^ curve
	**Poor**	**Favorable**	**%Correct**	
**1. Random forest model**				
(a) Internal test				0.90
Poor (*n* = 23)	18	5	78.3%	
Favorable (*n* = 99)	17	82	82.8%	
(b) External test				0.84
Poor (*n* = 42)	31	11	73.8%	
Favorable (*n* = 160)	27	133	83.1%	
**2. Rater #1**				
(a) Internal test				0.73
Poor (*n* = 23)	12	11	52.2%	
Favorable (*n* = 99)	6	93	93.9%	
(b) External test				0.78
Poor (*n* = 42)	28	14	66.7%	
Favorable (*n* = 160)	17	143	89.4%	
**3. Rater #2**				
(a) Internal test				0.75
Poor (*n* = 23)	13	10	56.5%	
Favorable (*n =* 99)	6	93	93.9%	
(b) External test				0.78
Poor (*n* = 42)	28	14	66.7%	
Favorable (*n* = 160)	18	142	88.8%	

* *ROC, receiver operating characteristic*.

## Discussion

In this article, we find that age, ventilated breathing status, WFNS grade, and Fisher grade are the independent predictors of poor outcome at discharge after rupture of ACoA aneurysms. A random forest machine learning model is developed for the prediction of outcome after rupture of ACoA aneurysms, and this model presents good prediction performance with areas under the ROC curves of 0.90 for the internal test and 0.84 for the external test.

Although a few studies (26, 27) show that the clinical outcome at 3 months is comparable between the patients in the clipping and coiling groups, endovascular coiling is recommended for patients suitable to both endovascular coiling and neurosurgical clipping, especially for posterior circulation aneurysms (28). The study of the International Subarachnoid Aneurysm Trial (4) reports that 26.1% of patients in the endovascular treatment group were dead or dependent at 2 months compared with 36.9% of the patients in the clipping group. A meta-analysis assesses evidence regarding safety and efficiency of endovascular clipping compared with coiling from 27 studies and finds that coiling yields a better clinical outcome (29). As to the clinical outcome after rupture of ACoA aneurysms instead of all aneurysms, a corresponding report is rare. In our institution, we mainly follow the recommendations (28) for surgical and endovascular methods of treatment of ruptured cerebral aneurysms from the American Heart Association and American Stroke Association, and find that a higher percentage of patients after rupture of ACoA aneurysms had poor outcome at discharge in clipping group than in coiling group (17.8 vs. 10.3%).

Many factors are associated with clinical outcome after aneurysmal SAH. Fisher grade (10) is by far the best known system of classifying the amount of SAH on CT scans and is proven to be a valid prognostic factor (4, 30). Increasing age is shown to be an independent predictor of poor outcome in aneurysmal SAH patients (7). GCS indicates patients' clinical status and is commonly used for assessment of impairment of consciousness level in response to defined stimuli. The WFNS grading system uses GCS and the presence of focal neurological deficits to evaluate the severity of SAH. A previous study proved that poor WFNS grade at presentation and advanced age are predictive of poor clinical outcome after endovascular coiling treatment (31). Although aneurysm morphological parameters are significantly related to aneurysm rupture (14, 32, 33), we find that none of the investigated aneurysm morphological parameters are independent risk factors of clinical outcome after rupture of ACoA aneurysm. When an aneurysm ruptures, it often causes bleeding in the brain. Bleeding is irrelevant with respect to aneurysm morphology once an aneurysm ruptures and may lead to serious health problems, such as SAH, hemorrhagic stroke, hydrocephalus, vasospasm, coma, and short-term or permanent brain damage (34). The prognosis of patients with a ruptured aneurysm depends on the patient's age, general health status, and neurological conditions. As discussed previously, Fisher grade and GCS are valuable score systems to evaluate one's neurological conditions (10, 31). Therefore, it is not surprising that none of the investigated aneurysm morphological variables is an independent predictor of clinical outcome after rupture of ACoA aneurysm.

A few models have been developed to predict the outcome after rupture of aneurysms. de Toledo et al. (6) construct a model using only two attributes (WFNS and Fisher's scale) with a C4.5 algorithm from a cohort of 634 patients; an accuracy of 73% was achieved for poor outcome prediction in their external validation. Recently, Hostettler et al. (35) perform decision tree analysis in aneurysmal SAH for prediction of short-term clinical outcome from a cohort of 548 patients; it is found that the prediction accuracies for good and poor outcomes were 66.7 and 75.4% in two testing data sets, respectively. Both the above two studies deal with intracranial aneurysms at different locations. We develop a random forest model to predict clinical outcome after rupture. It is noted that our study only focuses on ACoA aneurysms from a cohort of 809 patients, and aneurysm morphological information is also considered. Our model achieves good performance with accuracies of 78.3 and 73.8% for predicting poor outcome in internal and external tests, respectively. We also compare the prediction performance between our model and two raters. Although the specificity of the random forest model is lower than that of the raters, the sensitivity of the model is significantly superior to that of the raters (i.e., our machine learning model is especially useful in predicting poor outcome). Therefore, our machine learning model can be used to detect those who will have a poor outcome. Rupture of aneurysms is an acute neurological disease and determining its prognosis is crucial for both patients' relatives and clinicians. Selecting those who will have a poor outcome from a machine learning model is useful for clinicians to establish proper treatment and management strategies.

## Limitations

There are several limitations in this study. First, this is a single-center study, and only the Chinese population is involved, which may limit generalization to other centers. Second, we only investigate patients' short-term outcome, and their long-term clinical outcome is not evaluated. Moreover, as a retrospective study, clinical outcome evaluation may be biased, which may influence the application of the random forest model to the external validation data set. Finally, the random forest model has not been externally validated using data sets from other institutions. Nevertheless, we focus on ACoA aneurysms and the number of ruptured ACoA aneurysms is large; the developed random forest model presents good performance in the outcome prediction, which might aid in clinical decision making.

## Conclusions

In summary, we investigate the risk factors of clinical outcome after rupture of ACoA aneurysms in this study. It is shown that poor outcomes are significantly associated with patients' age, breathing status, WFNS grade, and Fisher grade. The aneurysm morphological parameters are not independent predictors. A random forest machine learning model is developed for the outcome prediction. The developed model is further validated using internal and external testing data sets, and good prediction performance is achieved.

## Data Availability Statement

All datasets generated for this study are included in the article/Supplementary Material.

## Ethics Statement

The studies involving human participants were reviewed and approved by The Medical Ethics Committee of The First Affiliated Hospital of Wenzhou Medical University. Written informed consent for participation was not required for this study in accordance with the national legislation and the institutional requirements.

## Author Contributions

BZ, XC, YY, and JL contributed to the study conception and design. YY acquired the funding. CZ, YC, YD, XJ, LL, BL, and NX collected the patients' data. JC, YX, and CC checked the data. JL and NX performed statistical analyses and drafted the article. YY and JL critically revised it. All authors reviewed the final manuscript and approved it to be submitted.

## Conflict of Interest

The authors declare that the research was conducted in the absence of any commercial or financial relationships that could be construed as a potential conflict of interest.
