# Supplementary material for: Prediction of Clinical Outcome at Discharge After Rupture of Anterior Communicating Artery Aneurysm Using the Random Forest Technique
Source: Front Neurol. 2020 Oct 29;11:538052. doi: 10.3389/fneur.2020.538052 (PMC7658443; doi:10.3389/fneur.2020.538052)
Supplement: Supplementary file 1 [file Data_Sheet_1.docx]

Supplementary Material

# Supplementary Figures and Tables

## Supplementary Tables

**Table S1:** Baseline characteristics for external independent population

| **Variables** | **Good outcome (n=160)** | **Poor outcome (n=42)** | **OR 95%CI** | **P value** |
| --- | --- | --- | --- | --- |
| **Demographic** |  |  |  |  |
| Men | 77 (48.1%) | 27 (64.3%) | 0.81 (0.26-1.04) | .062 |
| Age (yr) | 56.1±12.9 | 59.6±13.1 | 1.02 (0.99-1.05) | .119 |
| **Medical history** |  |  |  |  |
| Hypertension | 101 (63.1%) | 26 (61.9%) | 0.95 (0.47-1.91) | .884 |
| Current smoking | 53 (33.1%) | 17 (40.5%) | 1.37 (0.68-2.76) | .373 |
| Coronary artery disease | 1 (0.6%) | 2 (4.8%) | 7.95 (0.70-89.89) | .110 |
| Previous stroke | 4 (2.5%) | 1 (2.4%) | 1.00 (0.11-9.20) | 1.0 |
| **Clinical examination** |  |  |  |  |
| Breathing status |  |  |  | <.001 |
| Spontaneous | 158 (98.8%) | 26 (61.9%) | 1.0(Referent) |  |
| Ventilated | 2 (1.3%) | 16 (38.1%) | 48.62(10.56-223.93) |  |
| Pupillary reactivity |  |  |  | <.001 |
| Reactive(at least unilaterally) | 158 (98.7%) | 32 (76.2%) | 1.0(Referent) |  |
| Unreactive | 2 (1.3%) | 10 (23.8%) | 24.69 (5.16-118.07) |  |
| **Neurological examination** |  |  |  |  |
| GCS | 14.2±2.1 | 9.3±4.6 | 0.69 (0.62-0.77) | <.001 |
| WFNS grade | 1.4±1.0 | 3.5±1.7 | 2.37 (1.85-3.03) | <.001 |
| **Radiological findings** |  |  |  |  |
| Fisher grade | 3.6±0.7 | 3.9±0.4 | 2.74 (1.24-6.07) | .001 |
| Multiple aneurysm | 17 (10.6%) | 5 (11.9%) | 1.29 (0.44-3.77) | .784 |
| **Treatment methods** |  |  |  | <.001 |
| Endovascular treatment | 99 (61.9%) | 20 (47.6%) | 1.0 (Referent) |  |
| Surgical treatment | 46 (28.7%) | 8 (19.0%) | 0.88 (0.36-2.15) |  |
| Conservative treatment | 15 (9.4%) | 14 (33.3%) | 4.62 (1.93-11.06) |  |

GCS, Glasgow coma score; WFNS, World Federation of Neurosurgical Societies.

## Supplementary Figures


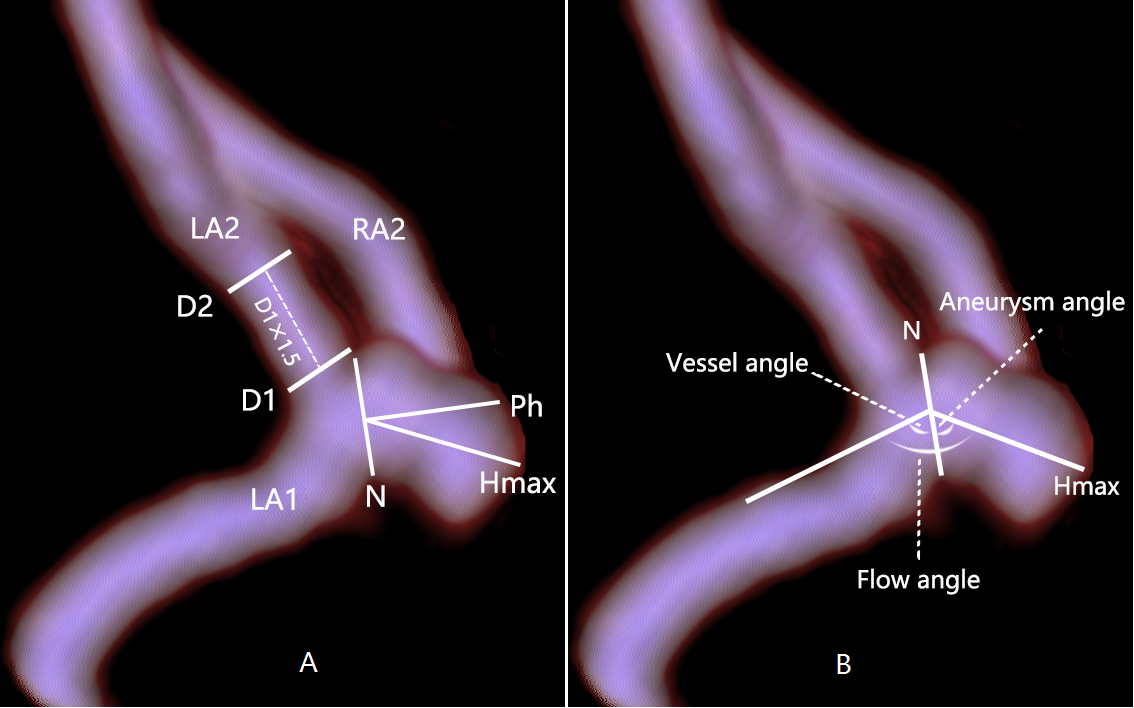


**FIG. S1** Measurement of aneurysm (A) sizes and (B) angles on CT angiography. *N*, neck size; *Hmax*, aneurysm height (the greatest distance from the center of aneurysm neck to the aneurysm dome); *Ph*, perpendicular height (the largest perpendicular distance from aneurysm neck to the aneurysm dome); vessel size, the average diameter of all arteries associated with the aneurysm; size ratio, the ratio of *Hmax* to vessel size; aspect ratio, the ratio of *Ph* to *N*.
